# Solitary Plasmacytoma of the Breast: A Rare Entity

**DOI:** 10.7759/cureus.53612

**Published:** 2024-02-05

**Authors:** Shiva Shiva, Kushagra Gaurav, Akshay Anand, Shivanjali Raghuvanshi, Abhinav A Sonkar

**Affiliations:** 1 Surgery, King George's Medical University, Lucknow, IND; 2 Pathology, King George's Medical University, Lucknow, IND

**Keywords:** rare breast tumors, oncology, breast cancer, plasma cell tumors, solitary plasmacytoma

## Abstract

Solitary plasmacytoma of the breast is an extremely rare neoplastic entity characterized by the localized proliferation of neoplastic plasma cells within the breast tissue that requires careful consideration due to its clinical and radiological resemblance to more common breast malignancies. While plasmacytomas are typically associated with multiple myeloma (MM), primary involvement of the breast as a solitary lesion is exceptionally rare. In this report, we present a case of solitary plasmacytoma of the breast in a 55-year-old female patient who presented with a palpable breast mass and no signs of systemic multiple myeloma. Our objective is to discuss the clinical presentation, radiological features, and histopathological findings and highlight the importance of comprehensive diagnostic workup and management planning for solitary plasmacytomas of the breast.

## Introduction

The malignant proliferation of plasma cells is termed plasmacytoma, which usually presents as a part of systemic multiple myeloma (MM). When located outside the bone marrow, it is termed extramedullary plasmacytoma, occurring most commonly in the upper airways [1,2]. Other sites of isolated extramedullary tumors are the gastrointestinal tract, lungs, pancreas, spleen, and, very rarely, the breast [1,2]. The incidence of breast plasmacytomas is approximately 1.5% of all the occurred plasmacytomas, nearly 15% of which are classified as primary, while the others are secondary to multiple myeloma [3]. Due to the rarity of the presentation, standard treatment guidelines are not available, but wide local excision with or without radiotherapy is recommended. Around 30%-50% of these patients develop multiple myeloma within 1.5-2.5 years, which requires further systemic therapy; hence, regular and short-term follow-up is essential [4].

## Case presentation

A 55-year-old postmenopausal, nonsmoker, nonalcoholic female with no comorbidities presented to the outpatient department with a history of a lump in her left breast for 20-25 years and ulceration of the overlying skin recently. There was no associated pain, nipple discharge, weight loss, fatigue, fever, bone pain, or systemic symptoms. There was no history of surgery, trauma, or radiation exposure to the breast. She denies any family history of breast, ovarian, or other malignancies. She attained menarche at the age of 14 and menopause 10 years prior to presentation. She had three children, all breastfed adequately. She denies taking birth control pills.

On examination, she was in good general condition with stable vitals and a Karnofsky score of 90. Systemic examinations were unremarkable. On local examination, a solitary, non-tender, firm, irregular-shaped lump of size 3×3 cm was palpable in the upper and lower lateral quadrants of the left breast without any attachment to the skin or underlying tissues, with no local rise in temperature and normal ipsilateral axilla. The contralateral breast and axilla were normal. An ulcer of size 1×1 cm was present near the lump in the upper outer quadrant with surrounding skin discoloration. It was provisionally diagnosed as adenocarcinoma of the breast, with malignant phyllodes, lymphoma, and fat necrosis as differentials.

**Figure 1:**
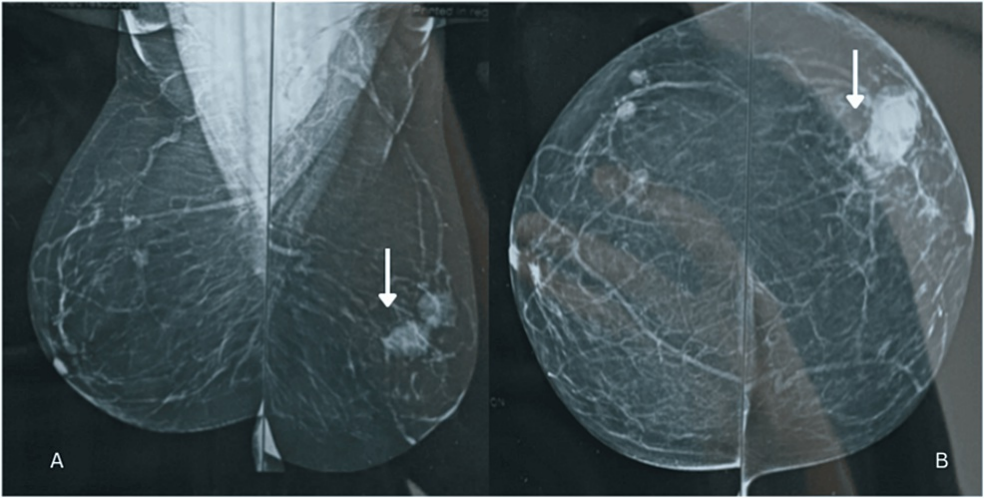
Mammography of B/L breast. A: MLO view, B: craniocaudal view Arrows: lesion in the left breast B/L: bilateral, MLO: mediolateral oblique

The chest X-ray did not reveal any abnormalities. Mammography of the left breast revealed a well-defined hyperdense lesion with circumscribed margins measuring 21×23×21 mm, 36 mm distant from the nipple-areola complex (NAC) in the upper outer quadrant (Figure 1).

**Figure 2:**
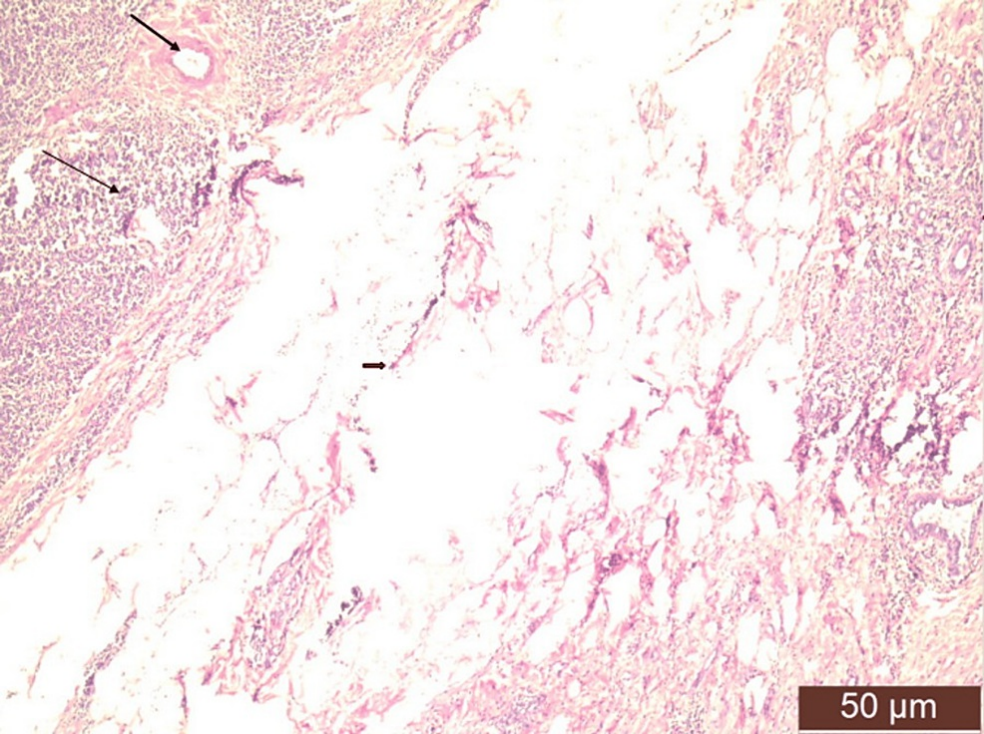
Photomicrograph showing infiltration of atypical plasma cells (thin arrow) in breast parenchyma. Adjacent breast parenchyma is seen (thick arrow) along with mammary fat (arrowhead).

**Figure 3:**
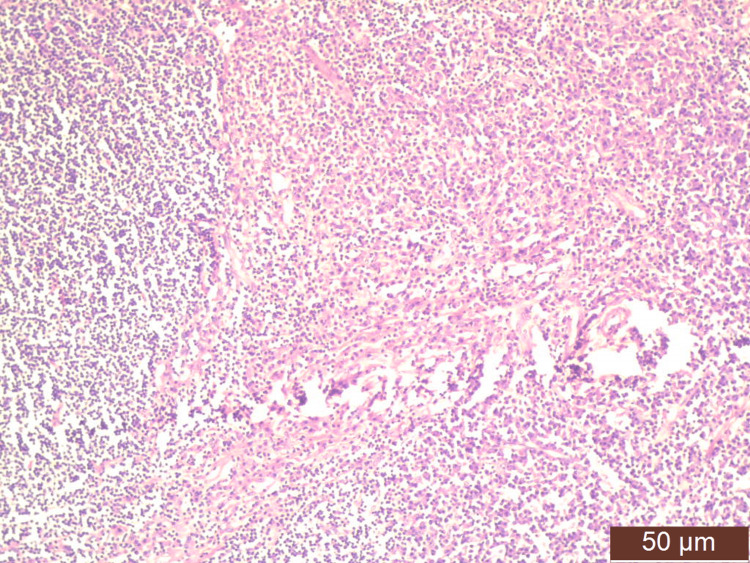
Infiltration of atypical plasma cells (H&E (low power)). H&E: hematoxylin and eosin

On high-resolution ultrasound (HRUSG) correlation, the above lesion corresponded to a well-defined, circumscribed, oval, hypoechoic, wider-than-taller lesion of size 20×21×15 mm, which showed mild vascularity on the color Doppler with few internal calcifications at the 2-3 o'clock position with few strands reaching up to the skin, causing its thickness to be approximately 3.2 mm (Breast Imaging-Reporting and Data System (BI-RADS) V). Another similar lesion of size 21×21×22 mm, 45 mm from the NAC, was also noted in the upper outer quadrant, most likely a satellite lesion. The left axilla showed a few enlarged lymph nodes, with the largest measuring 20×12 mm. Mammography of the right breast showed a well-defined, hyperdense lesion with circumscribed margins and peripheral calcifications, likely to be an intramammary lymph node. A cytological examination of a smear taken by fine-needle aspiration showed a lymphomatous lesion. Histopathological examination of the incisional biopsy (core cut biopsy) from the mass showed the presence of primary follicles, interfollicular areas infiltrated by a large number of plasma cells, and few lymphocytes suggestive of plasmacytoma (Figures 2, 3).

**Figure 4:**
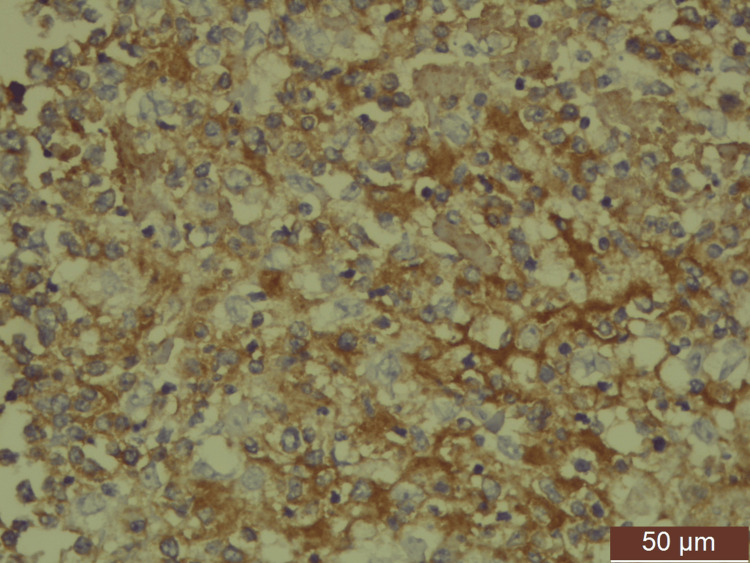
Atypical plasma cells show membranous positivity of CD138 (IHC (high power)). IHC: immunohistochemistry

**Figure 5:**
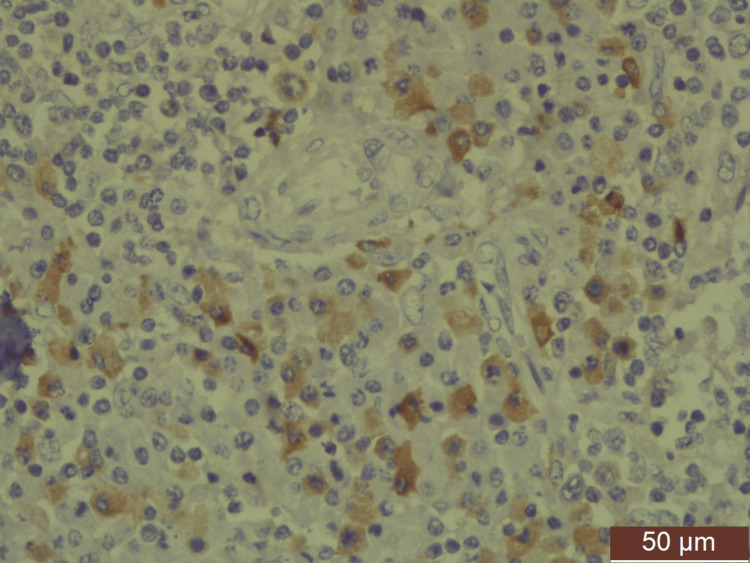
Atypical plasma cells are Kappa light chain-positive (IHC (high power)). IHC: immunohistochemistry

**Figure 6:**
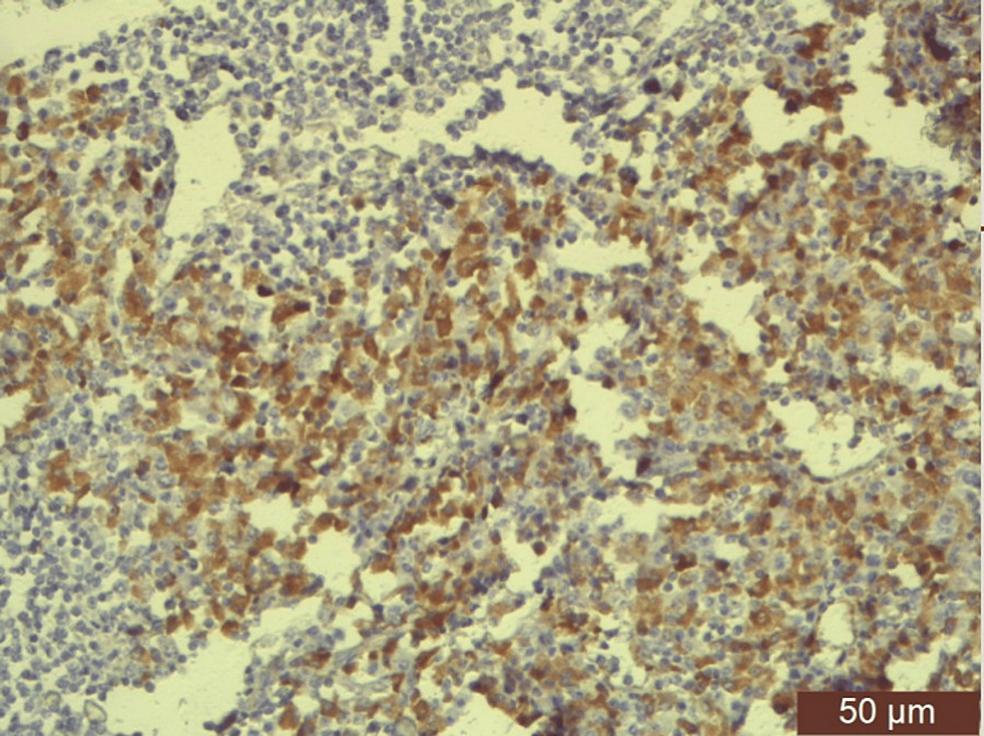
Atypical plasma cells show a high Ki67 index. Ki67 is nuclear positive (50%-60%).

Immunohistochemistry showed the following results: CK, negative, suggesting a non-epithelial origin; CD138, positive (Figure 4), with Kappa-positive (Figure 5) and Lambda-negative cells, indicating monoclonal origin of plasma cells; Ki67, 50%-60% (Figure 6); CD3 and vimentin, positive in intermittent T lymphocytes; CD20 and BCL2 positivity in follicles; CD10, inconclusive; and CD34, negative.

A complete workup was done to rule out multiple myeloma. The liver function test, renal function test, lactate dehydrogenase, serum electrolytes, bicarbonate, and calcium levels were normal. Pelvic X-rays and abdominopelvic ultrasonography were also normal. The serum immunoglobulin profile was within normal limits, except for mildly elevated IgG levels. Free Kappa, Lambda, and their ratios were within normal limits. Plasma protein electrophoresis was normal with an absent M band. Serum immunofixation electrophoresis showed no monoclonal gammopathy. Histology of bone marrow aspirate revealed 5% plasma cells (mainly small mature form). An 18-fluorodeoxyglucose positron emission tomography (FDG-PET) scan showed a hypermetabolic lesion in the outer quadrant of the left breast along with hypermetabolic left axillary lymphadenopathy, with no other lesion elsewhere in the body.

She was then diagnosed as having a solitary plasmacytoma of the breast without metastasis. She underwent wide local excision with rotational mastopexy and axillary dissection.

## Discussion

Plasmacytomas are tumors of plasma cells. The diagnosis of solitary extramedullary plasmacytoma is made when there is a single extramedullary mass of clonal plasma cells with a normal bone marrow biopsy and skeletal survey in the absence of anemia, hypercalcemia, or renal impairment due to plasma cell dyscrasia and an absent or low serum or urinary level of immunoglobulin.

Breast plasmacytomas are rare tumors; their diagnosis becomes relatively easy when they occur with MM because of the high level of suspicion. On the other hand, isolated breast plasmacytomas are difficult to diagnose as the usual presentation is a breast lump, which resembles other benign or malignant breast diseases. Most of the time, it is diagnosed incidentally on histopathological examination. A complete workup should be done to rule out multiple myeloma after the diagnosis of isolated plasmacytoma, as was done in the index case.

**Figure 7:**
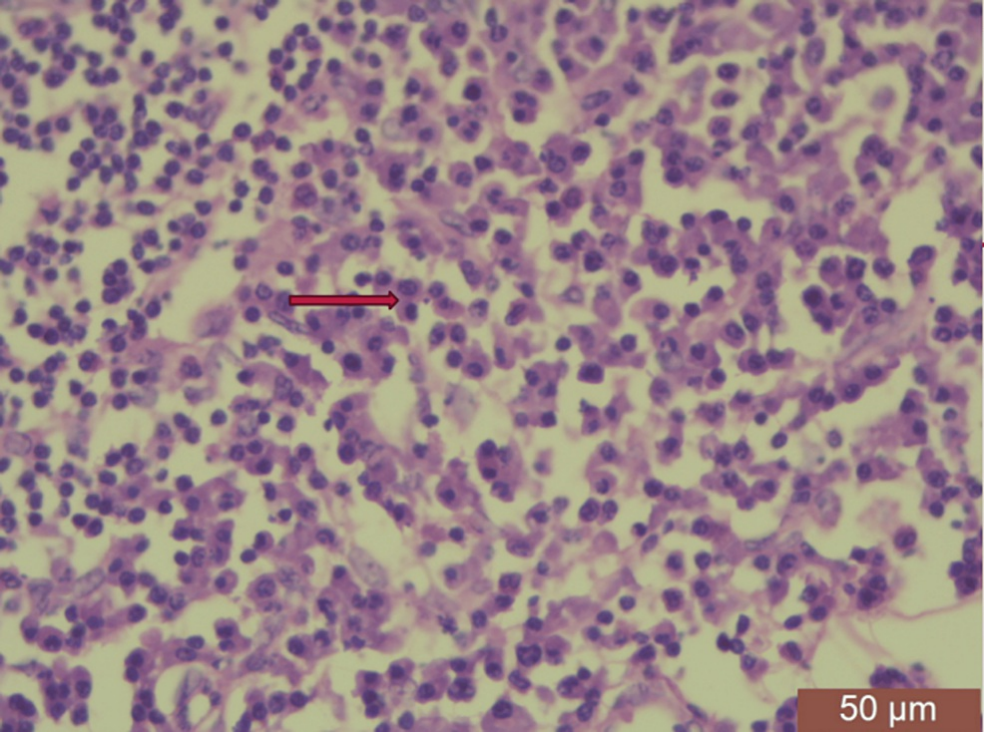
Atypical plasma cells displaying eccentric nucleus and a moderate amount of cytoplasm. Arrow: binucleate cells

Because of the similarity in clinical presentation with adenocarcinoma, it becomes very important to differentiate between radiology and histopathology. On histopathology, plasmacytoma shows atypical plasma cells containing irregular nuclei and prominent nucleoli in association with mature plasma cells (Figure 7).

Marrow plasma cells usually make up less than 5% of marrow cells. A literature review suggests that radiological features are not very characteristic and often resemble other benign and malignant breast diseases such as fibroadenomas, fat necrosis, abscesses, phyllodes tumors, lymphomas, and adenocarcinomas. However, magnetic resonance imaging (MRI) is found to be slightly better than ultrasonography (USG) and mammography [4]. The findings in the index case were in accordance with the available data.

Solitary extramedullary plasmacytomas are highly radiosensitive tumors [5]; hence, definitive radiotherapy is given for sites inaccessible to surgery with local and distant recurrence rates of 5% and 30%, respectively. The usual dose is 40-50 Gy in 20 fractions, with tumors larger than 5 cm requiring 50 Gy in 25 fractions [5]. The usual treatment for primary breast plasmacytoma is surgical excision, followed by adjuvant radiotherapy if margins are positive [6]. Adjuvant or definitive chemotherapy is reserved for aggressive diseases such as large tumors (>5 cm in diameter), high-grade tumors, and relapsed or refractory disease [7].

Alexiou et al. conducted a review of the literature and compared the outcomes of plasmacytoma patients treated with surgery alone, radiotherapy alone, and combined surgery and radiotherapy. They concluded that for upper aerodigestive tract plasmacytomas, overall and disease-free survival is better with the combined approach, while for plasmacytomas elsewhere in the body, recurrence rates are similar with all three approaches [5].

The recurrence rate is around 25%, warranting the need for chemotherapy or radiotherapy. Metastasis is rarely seen. A 10-year disease-free survival is reported to be around 70% [6]. In the absence of established guidelines for monitoring this rare disease, a tailored approach to follow-up is crucial. It is recommended that patients undergo regular follow-up visits with their healthcare provider to facilitate ongoing assessment and early detection of relapse or progression. The frequency and scope of these visits should be individualized, taking into account both the patient's comfort and the physician's judgment.

Allogeneic stem cell transplantation and intensive multi-agent therapies are other modalities that have been tried. However, because of the disease's rarity, concrete evidence is lacking for the treatment of breast plasmacytoma [3].

## Conclusions

To conclude, solitary breast plasmacytoma is a rare disease that needs careful clinical, radiological, and histopathological examination. Once diagnosed, after ruling out multiple myeloma and metastatic disease, surgical treatment with or without radiotherapy should be offered. Recurrence is rare, but these patients are at high risk of progression to multiple myeloma; hence, they should be kept for close follow-up.
